# Micro-RNAs Are Related to Epicardial Adipose Tissue in Participants With Atrial Fibrillation: Data From the MiRhythm Study

**DOI:** 10.3389/fcvm.2019.00115

**Published:** 2019-08-14

**Authors:** Khanh-Van Tran, Jordan Majka, Saket Sanghai, Mayank Sardana, Darleen Lessard, Zachary Milstone, Kahraman Tanriverdi, Jane E. Freedman, Timothy P. Fitzgibbons, David McManus

**Affiliations:** ^1^Division of Cardiovascular Medicine, Department of Medicine, University of Massachusetts Medical School, Worcester, MA, United States; ^2^Department of Biochemistry and Molecular Biology, Clark University, Worcester, MA, United States; ^3^Department of Quantitative Health Sciences, University of Massachusetts Medical School, Worcester, MA, United States

**Keywords:** epicardial adipose tissue, microRNA, atrial fibrillati, cardiac remodeling, inflammation

## Abstract

**Introduction:** Epicardial adipose tissue (EAT) has been linked to incidence and recurrence of atrial fibrillation (AF), but the underlying mechanisms that mediate this association remain unclear. Circulating microRNAs (miRNAs) contribute to the regulation of gene expression in cardiovascular diseases, including AF. Thus, we sought to test the hypothesis that circulating miRNAs relate to burden of EAT.

**Methods:** We examined the plasma miRNA profiles of 91 participants from the miRhythm study, an ongoing study examining associations between miRNA and AF. We quantified plasma expression of 86 unique miRNAs commonly expressed in cardiomyocytes using quantitative reverse transcriptase polymerase chain reaction (qPCR). From computed tomography, we used validated methods to quantify the EAT area surrounding the left atrium (LA) and indexed it to body surface area (BSA) to calculate indexed LA EAT (iLAEAT). Participants were divided into tertiles of iLAEAT to identify associations with unique miRNAs. We performed logistic regression analyses adjusting for factors associated with AF to examine relations between iLAEAT and miRNA. We performed further bioinformatics analysis of miRNA predicted target genes to identify potential molecular pathways are regulated by the miRNAs.

**Results:** The mean age of the participants was 59 ± 9, 35% were women, and 97% were Caucasian. Participants in the highest tertile of iLAEAT were more likely to have hypertension, heart failure, and thick posterior walls. In regression analyses, we found that miRNAs 155-5p (*p* < 0.001) and 302a-3p (*p* < 0.001) were significantly associated with iLAEAT in patients with AF. The predicted targets of the miRNAs identified were implicated in the regulation of cardiac hypertrophy, adipogenesis, interleukin-8 (IL-8), and nerve growth factor (NGF) signaling.

**Conclusion:** miRNA as well as EAT have previously been linked to AF. Our finding that iLAEAT and miRNAs 155-5p and 302a-3p are associated suggest a possible direct link to between these entities in the development and maintenance of AF. Further research is needed to study causal relationships between these biomarkers.

## Introduction

Atrial fibrillation (AF) is the most prevalent heart rhythm disorder in the world, with nearly 7 million Americans and 34 million individuals globally affected by it (1–4). It is associated with heart failure, stroke, dementia, and poor quality of life (1). Clinical risk prediction scores, including CHA_2_DS_2_VASC, perform modestly well in predicting outcomes of AF but explain little about the mechanisms behind disease onset and progression and only explain a small proportion of observed risk. As such, it is important to examine the molecular processes that drive AF, as it may afford new biomarkers and therapeutic avenues.

MicroRNA (miRNA) is a class of small non-coding RNA that are endogenously produced and have important regulatory function. miRNAs have been implicated in structural and electrical remodeling that are central to the development of AF (5–9). In addition to their regulatory role, they serve as biomarkers of disease states, as they exist in the plasma with remarkable stability (10). Examining the plasma miRome has provided unique insight into the molecular processes that underlie the pathogenesis of AF.

Epicardial adipose tissue (EAT) is a layer of metabolically active adipocytes that lie between the visceral pericardium and the myocardium without fascial boundaries. Due to its close proximity, this adipose depot has paracrine and vasocrine effects on the myocardium and plays a critical role in the development and maintenance of AF (11–13). Several groups, including ours, have shown correlation between EAT and onset as well as severity of AF. Our group has previously shown that an indexed measure of EAT surrounding the left atrium, called iLAEAT, is independently associated with the severity and recurrence of AF after catheter ablation (14).

Although much is known about the interaction of these biomarkers individually and their effects on the pathogenesis of AF, the relationship between EAT and plasma miRNAs has not been studied before. Thus, we hypothesized that increasing levels of EAT measured on computed cardiac tomography are associated with expression of circulating plasma miRNAs and tested this using data from a prospectively recruiting, contemporary cohort of AF patients.

## Methods

### Study Population

As part of the miRhythm study examining links between circulating miRNAs and AF, 584 participants were recruited at the University of Massachusetts Medical Center (UMMC) between April 2011 to July 2017 (15). Study protocol was approved by the University of Massachusetts Institutional Review Board (IRB #14875). Written consent was obtained from all participants to analyze pre-ablation miRNA expression. Of these, 91 participants underwent cardiac CT scan for evaluation of pulmonary venous anatomy prior to catheter ablation. These participants were included in our analysis. Participant information, including demographic, clinical, and baselines laboratory data, were abstracted from the UMMC AF Treatment Registry and hospital medical records by trained staff.

### EAT Measurements

EAT was measured using validated methods (16). CT scans were performed by technicians at UMMC with the Siemens Somatom Definition Flash 128 dual source CT scanner. Unenhanced CT scans were taken with a standardized FLASH protocol. Enhanced scans were performed by injecting contrast dye and scanning using sequential acquisition. Calculation of contrast timing was done by giving the patient a 15 mL contrast test bolus followed by a 50 mL saline injection at 5–6 mL/s. Acquisition times were held constant between 1 and 2 s. EAT was measured by scanning the heart in a four-chamber view, with the bottom of the heart in front view. Locations of EAT around the right and left ventricles and the left atrium were traced by identifying regions of CT scans within a threshold of−43 Hounsfield units (HU) for unenhanced CT scans and−15 HU for enhanced CT scans. The inter and intra-observer reproducibility for EAT measurement was (*r* = 0.89 and 0.95), respectively. EAT was indexed to body surface area and reported as iLAEAT.

### Echocardiographic Measurements

Complete 2D echocardiograms were performed during hospitalization. Linear dimensions and 2D volumes were measured according to ASE guidelines (17). We quantified left atrial (LA) volume), left ventricular end-diastolic (LVIDd) and end-systolic (LVIDs) dimensions, posterior wall thickness (PWT), interventricular septum thickness at end–diastole (IVSTd), left ventricular (LV) mass and left atrial function index (LAFI). LV mass was calculated by LV mass = 0.8 (1.04 [LVID + PWTd + SWTd]^3^ – [LVID]^3^) + 0.6 g (18). LAFI was calculated by the previously validated formula: LAFI = LA emptying fraction^*^LVOT-VTI / LAESVI (19).

### MiRNA Identification and Profiling

Ten cc of venous blood was obtained for study purposes after routine femoral venous sheath placement for catheter ablation of AF. This blood was processed to isolate plasma and stored as previously described (20). Briefly, blood was collected into blood collection tubes with solution containing sodium citrate. Samples were then centrifuged at 2,500 g for 22 min at 4°C. Plasma was separated from the cells and frozen at 80°C within 90 min of draw.

For this study, we selected 86 plasma miRNAs based on our RNAseq experiments done on 20 participants with cardiovascular disease and 20 without cardiovascular disease from the Framingham Heart Study Offspring 2 cohort (8th visit). We selected top expressed miRNAs from these 40 individuals for study in our RT-qPCR experiments (20). For all experiment, RNA was extracted from 200 μL of plasma. All methods used for cDNA synthesis, pre-amplification, and qPCR were performed according to Qiagen miScript Microfluidics Handbook by using Qiagen miScript Assays by the High-Throughput Gene Expression & Biomarker Core Laboratory at the University of Massachusetts Medical School (21). RNAs were isolated, and RT reactions performed by miScript II RT kit (Cat. No: 218161, Qiagen, Fredrick, MD, USA). miScript Microfluidics PreAMP Kit (Cat. No: 331455, Qiagen, Fredrick, MD, USA) was used for preamplification reactions. qPCR reactions were run using on BioMark System. BioMark System can detect single miRNA copy at 26–27 Cq compare to conventional qPCR platforms 36–37 (22).

We normalized the volume of plasma did not employ further methods of normalization in our dataset such as global mean normalization. Global mean is not ideal with our modest sample size and there are no reliable “housekeeper” gene to normalize miRNAs in the plasma (20). We believe that our Ct values are representative of relative concentrations. Differentially expressed miRNAs were analyzed using miRDB, an online database that captures miRNA and gene target interactions (23, 24). Network and functional analyses were generated through the use of Qiagen's Ingenuity Pathway Analysis (25).

### Statistical Analyses

Participants were divided into tertiles based upon iLAEAT values, placing them into either low (iLAEAT 0.08–0.59, *n* = 30), intermediate (iLAEAT 0.6–1.079, *n* = 31), or high (iLAEAT 1.08–3.58, *n* = 30) groups. Tertiles were created to identify participants at the greatest clinical risk for AF burden and recurrence. We examined the relationship between demographic, clinical, and echocardiographic variables and tertiles of iLAEAT using either χ^2^-squared tests for categorical variables or ANOVAs for continuous variables. A *p*-value of 0.05 was used as the standard significance threshold for statistical tests.

Pre-ablation miRNA expression was compared to iLAEAT using a series of linear regression models. Covariates included in the models were identified based upon characteristics that were determined to be significantly different between iLAEAT tertiles and included age, hypertension, heart failure, and posterior wall thickness. Bonferroni correction was also applied to account for multiple tests.

## Results

**Figure 1:**
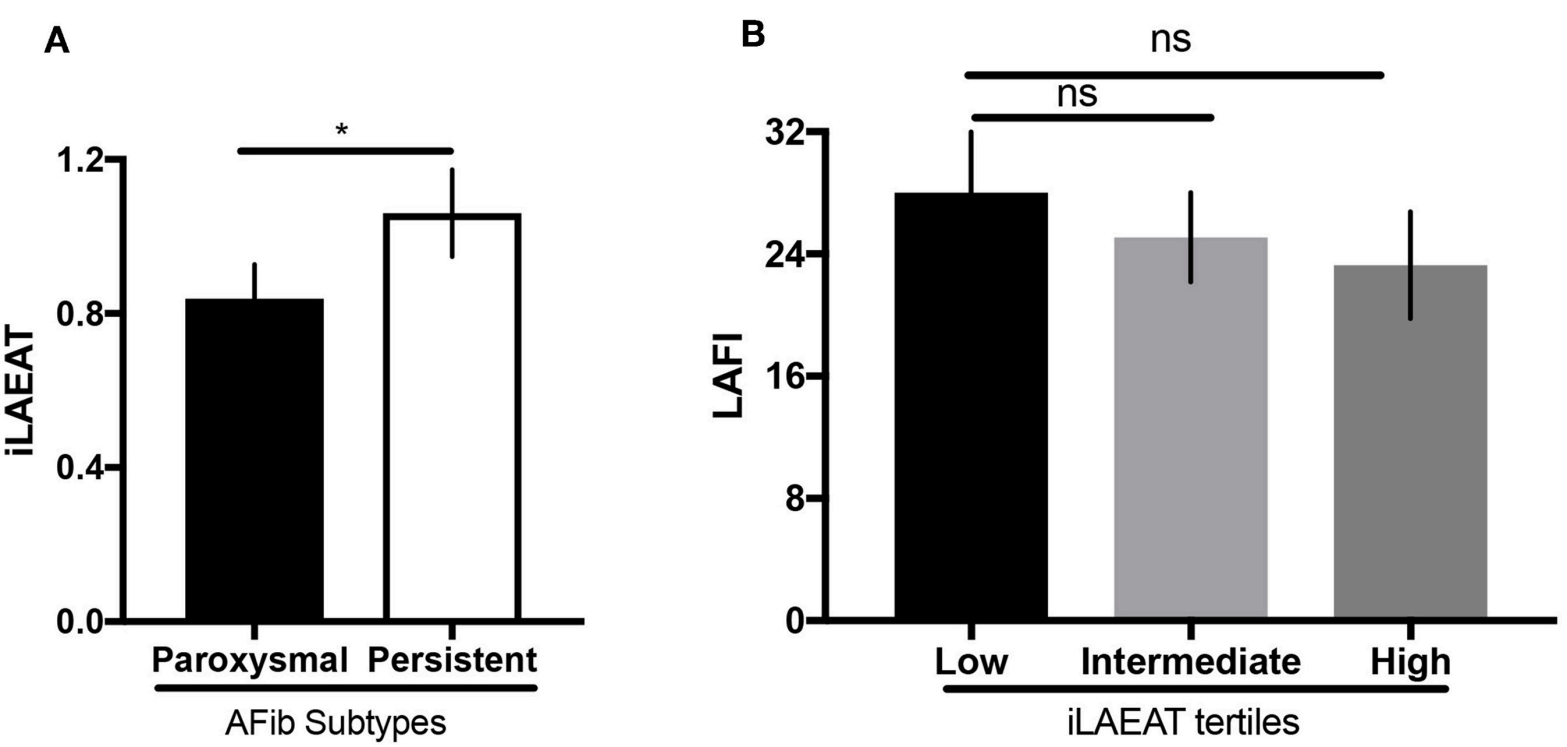
iLAEAT and left atrial function. **(A)** iLAEAT is higher in participants with persistent AF. **(B)** LAFI tended to be lower in patients with higher iLAEAT tertile. iLAEAT and LAFI data were available for 71 participants in our cohort (26 for low iLAEAT, 23 for intermediate iLAEAT, and 22 for high iLAEAT). Statistical analysis was performed using unpaired student *t*-test for AF subtypes (*p < 0.05) and one-way ANOVA analysis for iLAEAT tertiles. Error bars represent SEM. Panel **(A)** is adapted from work by Sanghai et al. (14).

We present participant characteristics based upon iLAEAT tertiles in Table 1. Participants almost exclusively identified as Caucasian (97%), and the majority were male (65%). Participants in the highest tertile of iLAEAT tended to be older, more likely to have a history of hypertension (86.7%, *p* = 0.01) and heart failure (26.7%, *p* = 0.01) and higher CHA_2_DS_2_-VASc scores (*p* = 0.04), thicker posterior walls (*p* = 0.01), and longer PR durations than did participants in the lower two tertiles. Participants with persistent AF have higher iLAEAT (Figure 1). As previously observed, participants who have higher iLAEAT have a higher likelihood of having persistent AF (14). Furthermore, there is a non-significant association in decreasing LAFI with increasing iLAEAT tertile (Figure 1).

**Table 1:** Characteristics of study patients divided into iLAEAT tertiles.

**Variable**	**Low iLAEAT**	**Intermediate iLAEAT**	**High iLAEAT**	***P*-value**
	**(0.08–0.59)**	**(0.6–1.079)**	**(1.08–3.58)**	
Age (years)	55.9 ± 10.2	59.8 ± 9.8	61.2 ± 7.9	0.08^**^
Male sex *n* (%)	15 (50.0)	22 (71.0)	22 (73.3)	0.11
Body mass index (kg/m^2^)	30.7 ± 6.8	32 ± 5.9	31.5 ± 5.2	0.72
**MEDICAL HISTORY**				
CHA2DS2-VASc score	1.7 ± 1.3	2.2 ± 1.5	2.6 ± 1.1	0.04^*^
Smoking *n* (% reporting current/ex-smoker)	7 (23.3)	11 (35.5)	12 (40.0)	0.36
Diabetes mellitus *n* (%)	3 (10.0)	9 (29.0)	6 (20.0)	0.18
Hypertension *n* (%)	16 (53.3)	24 (77.4)	26 (86.7)	0.01^*^
Heart failure *n* (%)	0 (0.0)	5 (16.1)	8 (26.7)	0.01^*^
Stroke/TIA *n* (%)	3 (10.0)	0 (0)	2 (6.7)	0.22
**ELECTROCARDIOGRAPHIC CHARACTERISTICS**				
PR duration (msec)^a^	165.5 ± 39.3	185.7 ± 28.3	172.7 ± 27.1	0.09^**^
QRS Duration (msec)^b^	91.3 ± 20.9	94.3 ± 21.6	91 ± 15.7	0.81
QTc duration (msec)^c^	514 ± 140.9	481.4 ± 123.6	443.6 ± 68.8	0.1
**ECHOCARDIOGRAPHIC CHARACTERISTICS**				
LVEF (%)^d^	58.2 ± 2.5	60 ± 5.6	54.4 ± 8.6	0.15
LA volume (mL)	78.0 ± 27.5	86.9 ± 20.1	83.3 ± 18.4	0.39
LVIDd (mm) mean (SD)	48.9 ± 6.0	50.5 ± 5.7	46.8 ± 5.8	0.19
LVIDs (mm) mean (SD)	31.5 ± 6.3	33.3 ± 5.6	30.1 ± 6.2	0.19
PWT (mm) mean (SD)	9.7 ± 1.6	10.3 ± 1.6	11.0 ± 1.7	0.01^*^
IVSTd (mm) mean (SD)	10.2 ± 2.3	10.5 ± 1.7	11.2 ± 1.9	0.2
LV mass (g)	172.1 ± 47.2	200.1 ± 64.1	186.9 ± 52.3	0.17
**LABORATORY CHARACTERISTICS**				
BNP mean (pg/mL)^e^	106.8 ± 120.4	112.2 ± 140.0	116.0 ± 130.7	0.97
CRP mean (mg/mL)^f^	5.7 ± 9.7	4.4 ± 7.1	3.9 ± 3.2	0.71

BNP, B-Natriuretic Peptide; CRP, C-Reactive Protein; LA, Left Atrium; IVSTd, Interventricular Septum Thickness end-Diastole; LA AP diameter, Left Atrium Anterior-Posterior diameter; LV, Left Ventricle; LVIDd, Left Ventricular Internal Dimension at end-Diastole; LVIDs, Left Ventricular Internal Dimension at end-Systole; PWT, Posterior Wall Thickness; TIA, Transient Ischemic Attack.

a PR data were available for 79 of the participants (29 for low iLAEAT, 23 for intermediate iLAEAT, and 27 for high iLAEAT).

b QRS data were available for 80 of the participants (29 for low iLAEAT, 24 for intermediate iLAEAT, and 27 for high iLAEAT).

c QTc data were available for 73 of the participants (28 for low iLAEAT, 20 for intermediate iLAEAT, and 25 for high iLAEAT).

d EF data were available for 28 of the participants (11 for low iLAEAT, 9 for intermediate iLAEAT, and 8 for high iLAEAT).

e BNP data were available for 68 of the participants (27 for low iLAEAT, 21 for intermediate iLAEAT, and 20 for high iLAEAT).

f CRP data were available for 69 participants (28 for low iLAEAT, 22 for intermediate iLAEAT, and 19 for high iLAEAT).

* Denotes significance of p < 0.05 between tertiles.

** Denotes marginal significance of p < 0.1 between tertiles.

Results from qPCR analysis of 86 miRNAs are included in Supplemental Table 1. Analysis of miRNA expression level as well as regression models run between iLAEAT and Cq are illustrated in Table 2. After statistical correction for multiple tests, two miRNAs (miR-155-5p and−302a-3p) remained significantly associated with higher tertile of iLAEAT. Although, we do not know the source, potential targets or the mechanisms by which they are transported in the plasma, we believe that plasma miRNAs are derived from cellular sources. Therefore, analysis of miRNA downstream targets and pathways may reveal information regarding the molecular pathways that are activated.

**Table 2:** Associations between iLAEAT and miRNAs.

**MiRNA ID**	**b-coefficient**	**95% confidence intervals**		**Cq**	***p*-value**
miRNA-100-5p	−0.09256	−0.16559	−0.019533	19.08	0.013684
miRNA-122-5p	−0.07597	−0.12271	−0.029219	15.84	0.001798
miRNA-106b-5p	−0.06955	−0.13335	−0.005748	16.17	0.03305
**miRNA-155-5p**	**0.01584**	**0.00769**	**0.02399**	**12.98**	**0.00024**
miRNA-184	0.01587	0.00351	0.028234	5.67	0.01282
miRNA-192-3p	0.01578	0.00133	0.030232	4.78	0.032953
miRNA-199a-5p	0.01335	0.00091	0.025786	16.68	0.035761
miRNA-19a-3p	−0.08618	−0.15284	−0.019515	15.41	0.011985
miRNA-19a-5p	0.01881	0.007	0.030627	5.12	0.002358
miRNA-20a-5p	−0.07285	−0.13287	−0.012837	15.14	0.018026
miRNA-21-5p	−0.08711	−0.16238	−0.011839	15.01	0.02391
miRNA-218-5p	0.01153	0.00195	0.021105	10.47	0.01914
miRNA-221-3p	−0.08579	−0.15833	−0.013249	17.57	0.021091
miRNA-29a-3p	−0.07983	−0.14971	−0.00994	17.91	0.025736
**miRNA-302a-3p**	**0.02139**	**0.01078**	**0.032**	**6.59**	**0.00016**
miRNA-182-5p	0.0118	0.00287	0.020725	11.85	0.010358
miRNA-30a-3p	0.01191	0.00057	0.023248	15.27	0.039776
miRNA-320a	−0.06294	−0.12578	−0.000095	17.76	0.049664
miRNA-196b-5p	0.01095	0.00053	0.021373	13.32	0.039796
miRNA-483-5p	0.01716	0.00639	0.027934	11.56	0.002286
miRNA-491-3p	0.01845	0.00683	0.030061	6.19	0.002422
miRNA-576-5p	0.0181	0.00557	0.030625	17.2	0.005243
miRNA-589-3p	0.01454	0.00373	0.025357	10.29	0.009236
miRNA-589-5p	0.01385	0.00123	0.026474	4.77	0.032054
miRNA-92a-3p	−0.09228	−0.16487	−0.019692	13.8	0.013415
miRNA-30a-5p	−0.083	−0.15325	−0.012747	17.27	0.021215
miRNA-26a-5p	−0.07741	−0.13605	−0.018773	17.44	0.01036
miRNA-24-3p	−0.05787	−0.11243	−0.003314	17.55	0.037918
miRNA-126-3p	−0.08408	−0.14623	−0.021933	15.79	0.00868
miRNA-451a	−0.08118	−0.14626	−0.016095	9.84	0.015193
let-7b-5p	−0.0718	−0.13272	−0.0109	15.17	0.0215
let-7c-5p	−0.06978	−0.12978	−0.0098	16.75	0.02324

*Bolded miRNAs signify significant relationships after Bonferroni correction for p < 0.0005. Cq, quantitation cycle; miRNA, microRNA*.

**Figure 2:**
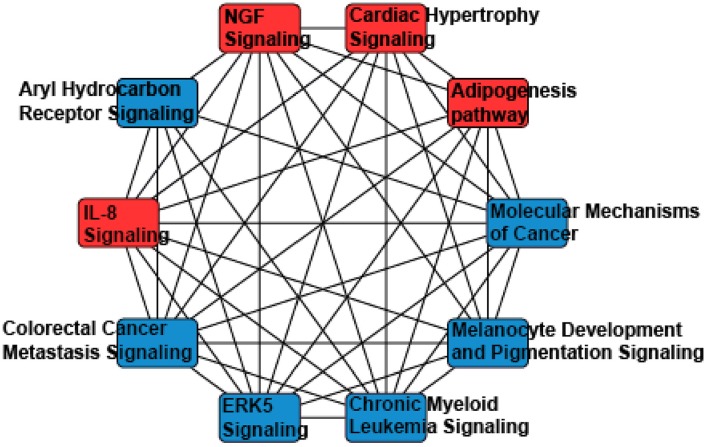
A network analysis of predicted targets of miR-155-5p and miR-302a-3p as performed by Ingenuity Pathway Analysis. Nodes represents signaling pathways, and lines are protein targets that are common between nodes. IL-8, Interleukin 8; NGF, Nerve Growth Factor; ERK5, Extracellular Related Kinase 5.

Thus, miRNAs downstream targets were analyzed using miRDB, an online database that captures miRNA and gene target interactions (23, 24). We do not have experimental data to guide selection of relevant downstream targets, and thus we included all predicted targets of miR-155-5p and−302a-3p in our analysis. As miRNA are known to act in concert, we used the combined targets of miR-155-5p and−302a-3p to perform further analysis (26). Ingenuity Pathway Analysis (IPA) was utilized to identify the molecular network and cellular toxicity pathways regulated by predicted targets. Canonical pathways were mapped to allow for visualization of the shared biological pathways through the common genes. The pathways identified and their associated genes are shown in Supplementary Table 2 and the top 10 significant pathways are displayed in Figure 2, including adipogenesis, cardiac hypertrophy, nerve growth factor (NGF), and IL-8 signaling.

## Discussion

Due to its proximity to the autonomic ganglia, myocardium, and coronary arteries, epicardial adipocytes can influence neighboring cells via both paracrine and vasocrine signaling (27). EAT has been implicated in the pathogenies of AF. We and others demonstrated previously that EAT is associated with incident and severity of AF (14, 28). miRNAs regulate the molecular processes that trigger the structural and electrophysiological remodeling key to the development of AF (6–9, 21). They are associated with incident and recurrent AF, and their dynamic nature after catheter ablation suggests that they are related to the pathogenesis of AF (5, 15, 21). In this work, we explore the relationship between iLAEAT and plasma miRNA and hypothesize their potential role in creating a substrate for AF.

We found two circulating miRNAs, miRNA 155-5p and 302a-3p, significantly associated with iLAEAT. Our pathway analysis of the downstream targets of miRNAs 155-5p and 302a-3p implicated them in the regulation of adipogenesis, cardiac hypertrophy, IL-8, and NGF signaling. In our cohort, increased iLAEAT is associated with increased posterior wall thickness. It is possible that miRNAs 155-5p and 302a-3p regulate pathways to increase formation of adipocytes and myocyte hypertrophy, as evident by echocardiographic phenotypes. It is unclear to us why in our dataset there is a significant association between iLAEAT and PWT and not IVSTd or LV mass. Perhaps, iLAEAT is in close proximity to the posterior wall and thus is able to exert mechanical stress or paracrine effects to cause local hypertrophy. Alternatively, we may be under-powered to examine the differences in IVSTd or LV mass in different tertiles of iLAEAT.

**Figure 3:**
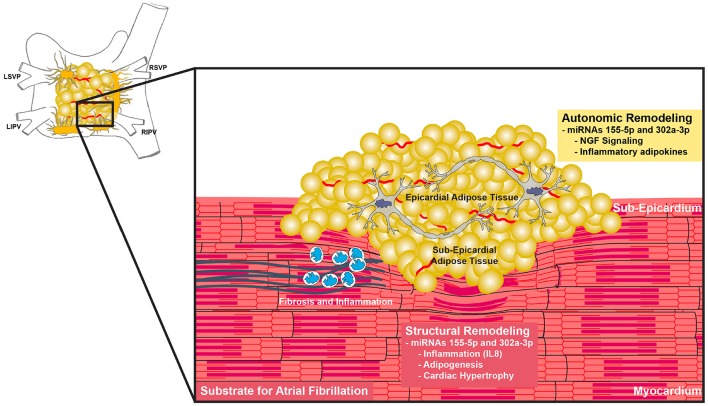
Proposed functions of miR-155-5p and miR-302a-3p. Increase EAT with concurrent upregulation of miR-155-5p and miR-302a-3p cause changes in adipokine secretion such as IL-8 and NGF. These changes lead to autonomic dysregulation and increased inflammation, creating a substrate for atrial fibrillation.

EAT has been shown to secrete adipokines that influence electrical remodeling (29). Our analysis predicts that miRNA 155-5p and 302a-3p regulate NGF signaling. There is much evidence suggesting that autonomic remodeling plays an important role in the pathogenesis of AF (30–32). The ganglionated plexi (GP), consisting of autonomic ganglia, are located on the epicardial surface of the heart and are typically surrounded by EAT (Figure 3) (33). Adipocytes have been shown to secrete NGF (34). NGF is up-regulated in the presence of AF and can induce the hyperactivity of GP (32, 35). In animal models, Yang et al. found that high plasma NGF levels create atrial substrate for AF and increase the incidence of inducible AF and its duration (36). Our analysis of miRNAs 155-5p and 302a-3p suggests a mechanism in which adipose tissue upregulates NGF to potentiate AF.

Several studies from the Sata laboratory have shown that EAT adipokine imbalance is a strongly linked of the development of atherosclerosis in men (37–39). Similarly, adipokines released from EAT may alter immune profiles the myocardium and surrounding tissue to increase inflammation and vulnerability to rhythm disturbances (40). Our results further support the hypothesis that EAT is an inflammatory mediator that is involved in adverse cardiac remodeling. EAT potentially contributes to inflammation and adverse structural remodeling via secretion of inflammatory adipokines such as IL-8 (40, 41). Circulating levels of IL-8 are associated to obesity-related factors such as BMI, waist circumference, C-reactive protein, interleukin (IL) 6, and tumor necrosis factor α (TNF-α) (42–44). IL-8, IL-6, IL-10, and TNF-α concentrations have been shown to be independently associated with AF (45). Patients with post-operative AF after coronary artery bypass graft surgery have higher serum IL-8 concentrations, indicating a role for inflammation in the development of AF post-surgery (46). miRNAs 155-5p and 302a-3p are implicated in the regulation of IL-8 and are associated with increased EAT, suggesting that secretion of IL-8 may be one of the ways in which EAT promotes inflammation to create a substrate for AF.

## Strengths and Limitations

We leveraged data from a prospectively enrolled contemporary cohort of participants with AF undergoing miRNA profiling, echo phenotyping as well as EAT quantification using cardiac CT. We analyzed plasma concentration levels of commonly expressed miRNAs in cardiomyocytes of a cohort of 91 patients undergoing CA and CT scans at UMMC.

Despite these study strengths, there are notable study limitations. Notably, we do not know the sources of the miRNAs, the mechanism by which they are found in the blood or if they are indeed involved in a signaling cascade. These biological questions are of great importance and will provide mechanistic insight. Unfortunately, they are beyond the scope of our study. Our modest sample size leaves for the possibility of falsely negative associations. This may explain why the negative association between iLAEAT and LAFI was not statistically significant. There is also a lack of diversity in patients sampled inhibits us from making generalizable claims. Furthermore, the associations made in this study between iLAEAT and miRNA expression are purely correlational. We have not tested a causal relationship between miRNAs 155-5p, 302a-3p and iLAEAT. Further experimentation at the bench is needed to elucidate the exact relationship between these miRNAs and EAT.

## Conclusion

In this study, we assessed the relationship between relative EAT area and plasma miRNA expression in patients with AF. We observed a significant, positive association between relative EAT area and miRNAs 155-5p and 302a-3p. Future validation and mechanistic studies are needed to improve our understanding of the pathological role of EAT in AF development.

## Data Availability

The datasets analyzed in this manuscript are not publicly available. Requests to access the datasets should be directed to david.mcmanus@umassmed.edu.

## Author Contributions

DM, TF, and JF supervised this work. DM, TF, K-VT, SS, JM, and MS contributed to hypothesis generation, conceptual design, and data analysis. KT, DL, and K-VT conducted experiments and analyzed data. All contributed to manuscript preparation.

### Conflict of Interest Statement

DM has received research grant funding from Bristol-Myers Squibb, Boeringher-Ingelheim, Pfizer, Samsung, Philips Healthcare, Biotronik, has received consultancy fees from Bristol-Myers Squibb, Pfizer, Flexcon, Boston Biomedical Associates, and has inventor equity in Mobile Sense Technologies, Inc. (CT). The remaining authors declare that the research was conducted in the absence of any commercial or financial relationships that could be construed as a potential conflict of interest.
